# A Novel Pipeline for Adrenal Gland Segmentation: Integration of a Hybrid Post-Processing Technique with Deep Learning

**DOI:** 10.1007/s10278-025-01449-y

**Published:** 2025-03-04

**Authors:** Michael Fayemiwo, Bryan Gardiner, Jim Harkin, Liam McDaid, Punit Prakash, Michael Dennedy

**Affiliations:** 1 School of Computing, Engineering, and Intelligent Systems, Ulster University, Londonderry, Northern Ireland UK; 2 Mike Wiegers Department of Electrical and Computer Engineering, Kansas State University, Manhattan, KS USA; 3 School of Medicine, National University of Ireland, Galway, Ireland

**Keywords:** CT segmentation, Adrenal gland, Image processing, Test-time augmentation

## Abstract

Accurate segmentation of adrenal glands from CT images is essential for enhancing computer-aided diagnosis and surgical planning. However, the small size, irregular shape, and proximity to surrounding tissues make this task highly challenging. This study introduces a novel pipeline that significantly improves the segmentation of left and right adrenal glands by integrating advanced pre-processing techniques and a robust post-processing framework. Utilising a 2D UNet architecture with various backbones (VGG16, ResNet34, InceptionV3), the pipeline leverages test-time augmentation (TTA) and targeted removal of unconnected regions to enhance accuracy and robustness. Our results demonstrate a substantial improvement, with a 38% increase in the Dice similarity coefficient for the left adrenal gland and an 11% increase for the right adrenal gland on the AMOS dataset, achieved by the InceptionV3 model. Additionally, the pipeline significantly reduces false positives, underscoring its potential for clinical applications and its superiority over existing methods. These advancements make our approach a crucial contribution to the field of medical image segmentation.

## Introduction

An adrenal gland consists of an inner core and an outer membrane, releasing essential hormones like cortisol, adrenalin and aldosterone into the human body. A hormonal imbalance brought on by excessive hormone production from the inner core or outer membrane manifests as specific clinical symptoms [1]. High-density resolution X-ray CT imaging can express the human adrenal gland, a vital endocrine organ. Computer models facilitate accurate delineation of the adrenal gland, enabling clinicians to identify abnormalities like nodules and support targeted interventions, providing valuable insights into the structural aspects through segmentation. This offers a complementary approach to biochemical tests, contributing to a comprehensive understanding of the adrenal gland’s condition. Clinicians typically manually draw the border of the adrenal gland, which takes time and has a significant subjective component. Segmenting the adrenal gland accurately presents a challenge due to its small size and the proximity of surrounding tissues, which often adhere closely to the greyscale and textured regions of interest. As a result, it is challenging to precisely segment the adrenal gland due to adhesion boundaries between the adrenal gland and its surrounding tissues [2]. Computer-based models have been used to segment different organs from medical images, but one of the main challenges is the ability to generalise when presented with unseen data. A large labelled training set consisting of captured radiological images and corresponding pixel-wise label maps is necessary for training a well-generalised deep learning model. The process of acquiring such a sizable labelled training set is resource intensive. The contouring of organs or tissues in 3D volumes necessitates laborious manual input and requires precise annotations of radiological images conducted first by skilled radiologists and then reviewed by other domain experts. On the contrary, obtaining large unannotated datasets of CT scans is considerably simpler. In recent years, there have been substantial scientific endeavours towards automating adrenal gland segmentation using CT scans and deep learning techniques have played a pivotal role.

A number of segmentation methods have been proposed to efficiently and accurately segment the adrenal gland in CT scans. For instance, to lessen class imbalance and computational load, Luo et al. [3] suggested an optimised two-stage cascaded deep neural network for adrenal segmentation on CT images. This network uses a localisation network in the pre-processing stage to identify the candidate volume of the target organ. A SmallorganNet model was created and trained with a boundary attention focal loss to further define the organ’s boundary within the screened volume. Using a patch-based network with random spatial initialisation, Tang et al. [4] also reported a 3D abdominal segmentation based on a statistical fusion of overlapping regions of interest (ROIs). The task of 3D abdominal segmentation included the segmentation of 12 structures in the abdomen that contained adrenal glands. Other related works include segmenting the adrenal model based on shape associating level set in a series of CT images and the semi-supervised 3D abdomen multi-organ segmentation via deep multi-planar co-training [2, 5].

Deep learning approaches, particularly CNN-based architectures like U-Net, have outperformed traditional methods in terms of accuracy and robustness [6]. Incorporating attention mechanisms and multi-modal information has further enhanced segmentation outcomes [7]. Adrenal gland segmentation is still an area of ongoing research and further exploration is needed to fine-tune existing strategies for specific medical imaging tasks and modalities. Recently, test-time augmentation (TTA) has emerged as a valuable technique in the realm of medical image segmentation. This approach involves applying various transformations to test images before feeding them into a trained segmentation model. It essentially provides the model with multiple perspectives of the same image, enabling it to capture a broader range of features and improve segmentation accuracy. One of the key advantages of TTA is its ability to enhance model robustness. By considering augmented versions of the test image, the model becomes more resilient to variations in image quality, orientation, or other factors that commonly occur in clinical practice. This is particularly crucial in medical imaging where data can be noisy or exhibit variability across different scans. Furthermore, as observed and reported in this study, TTA often leads to a reduction in false positives and false negatives. By incorporating diverse views of the same image, the model is better equipped to identify subtle structures or anomalies that might have been missed with a single input. TTA has been used to improve the segmentation performance in segmenting medical images like brain tumour [8], breast lesions [9], melanoma [10], prostate cancer [11], and pneumothorax [12]. However, to the best of our knowledge, the use of TTA for adrenal gland segmentation has not been reported in the literature.

While certain literature emphasises new architectures, algorithms, and models, this study devotes its attention to elucidating the principles, guidelines, approaches, and procedural steps that enhance segmentation outcomes with established architectures. Thus, it is imperative to clarify that the objective of this paper is not comparative analysis between models, but rather an in-depth exploration and assessment of the impact and significance of the pipeline when paired with the chosen architecture and its respective backbone. Hence, this paper presents a novel pipeline for segmenting both left and right adrenal glands. The pipeline was implemented using 2D nnUNet [6], TransUNet [13], and also UNet architectures on separate backbones namely VGG16, Resnet34, Inceptionv3, and the default UNet. The proposed pipeline provides a reliable and robust methodology for adrenal gland segmentation in CT, and the study demonstrates that the use of TTA in medical image segmentation is a promising technique that holds great potential for improving segmentation performance, especially in scenarios where robustness to variations in data is critical.

## Methods

### Experimental Data

A multimodal abdominal multi-organ segmentation benchmark called a multi-modality Abdominal Multi-Organ Segmentation challenge 2022 (AMOS) was made available on a large, clinically diverse scale [14]. With voxel-level annotations of 15 abdominal organs, including the right and left adrenal glands, it offers CT scans gathered from patients from many centers, vendors, modes, phases and diseases. The dataset comprises numerous DICOM images related to the same patient. Both the Longgang District Central Hospital (SZ, CHINA) and the Longgang District People’s Hospital (SZ, CHINA) provided the datasets. The published data for the chosen datasets include whole images in a nifty format. Additional data were obtained from the Multi-Atlas Labeling Beyond the Cranial Vault [15], which was used in the MICCAI2015 challenge, which contained thirteen abdominal organs, including the right and left adrenal glands.

In line with the scope of this study, 300 CT scans were used to segment the right and left adrenal glands. We used 200 CT scans for training, while 60 CT scans were used for validation, and the remaining 40 CT scans were held to test the developed models’ generalisation. Each CT scan had a different number of slices, ranging from 67 to 369, and the slices had pixels sizes of 512 × 512 and 768 × 768. To corroborate the results of our pipeline, we carried out the same experiments on new dataset from the Multi-Atlas Labeling Beyond the Cranial Vault, using 25, 5, and 2 CT scans for training, validation, and testing, respectively.

### Implementation Pipeline

**Fig. 1:**
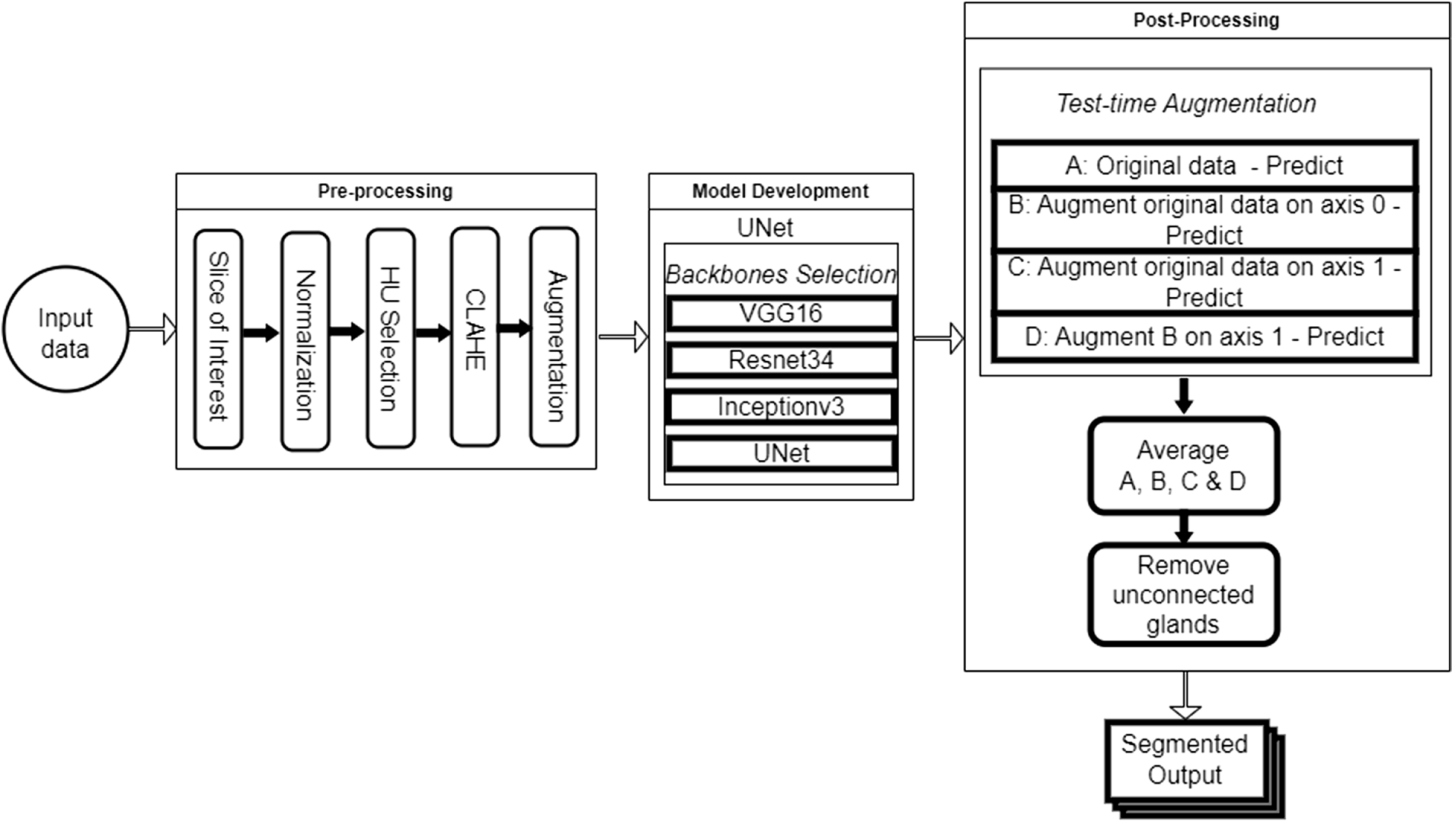
Overview of the proposed pipeline for adrenal gland segmentation, illustrating three key stages: (1) pre-processing, which includes data normalisation, clipping Hounsfield values, and applying CLAHE; (2) model development using the 2D UNet architecture with various backbones; (3) post-processing, involving test-time augmentation and removal of unconnected segmented regions to refine the final segmentation results

This section presents a detailed description of the proposed pipeline for adrenal gland segmentation on CT images. The nnUNet, TransUNet, and UNet architectures were explored, with the UNet specifically implemented using four different backbones: ResNet34, VGG16, InceptionV3, and the default UNet. Each adrenal gland was segmented independently using the UNet segmentation models built on the Keras deep learning framework and Tensorflow as the backend. As shown in Fig. 1, the proposed pipeline is divided into three unique processes: (1) pre-processing; (2) implementing the UNet model with various selected backbones; (3) post-processing. Each stage of the pipeline is discussed separately below.

### Pre-processing

The pre-processing stage plays a pivotal role in refining the raw CT image data prior to the segmentation of the adrenal gland, enhancing the efficacy and accuracy of the subsequent analyses. This section encompasses five crucial stages, each contributing to the optimisation of the dataset for robust segmentation outcomes.

#### Data Normalisation

The pixel-wise normalisation technique was used to accelerate model learning. The normalisation of an image consists of dividing each pixel value by the maximum value a pixel can have. A fully automated pre-processing pipeline was developed that normalises whole-body scans with pixel values ranging between 0 and 1.

#### Clipping the Hounsfield Values

While different Hounsfield range values have been recorded for segmenting adrenal glands [4], this study used a Hounsfield range of [10] for the selected CT scans, the selection range was based on the outcomes of different experiments conducted in this study.

#### Contrast Limited Adaptive Histogram Equalisation (CLAHE)

CLAHE is implemented to enhance local contrast, facilitating improved discrimination of structures within the CT scans. By transforming each pixel using a function taken from a nearby region, adaptive histogram equalisation (AHE) reorganises a skewed contrast distribution. The improved contrast-enhancing technique known as contrast limiting prevents noise amplification when contrast increases in CLAHE, a variation of AHE, which uses histogram features [16]. This takes care of the over-amplification of image contrast. While blocking the noise increment, CLAHE is used to enhance contrast quality in the vicinity of the adrenal glands.

**Fig. 2:**
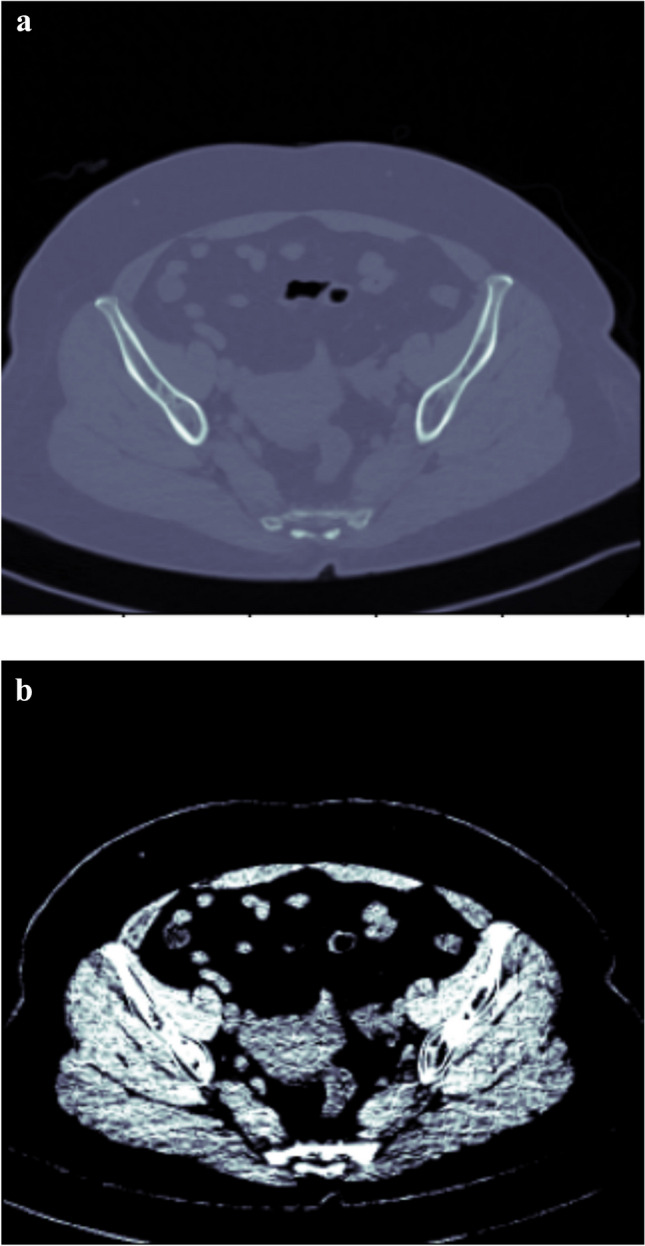
**a** Sample of a slice before pre-processing. **b** Sample of a slice after pre-processing

Figure 2a shows a slice sample before pre-processing and Fig. 2b shows the respective slice after applying the normalisation, CLAHE and clipping the Hounsfield values to (10, 60).

#### Selection of Slices of Interest

The selection of slices of interest, specifically those containing adrenal glands, was guided by the ground truth labels in the dataset and applied solely during the training phase. Out of 26,069 slices in the training dataset, only 1749 contained adrenal glands. Including the remaining 24,320 slices, which lacked adrenal glands, did not enhance the training process, as observed in preliminary experiments conducted to refine the methodology. As such, only slices containing adrenal glands were used during training to focus on relevant regions and optimise the model’s learning process. It is important to note that during testing, the developed model was evaluated on the complete test dataset, which included both slices with and without adrenal glands. This ensures the model’s applicability to real-world scenarios where no prior selection of slices is feasible.

#### Augment the Training Data

Augmenting the training data diversifies the dataset, augmenting the model’s ability to generalise and effectively adapt to varying anatomical configurations, ensuring robust segmentation performance. In the previous step, 24,320 slices from the 26,069 slices of the training data were removed; this drastically reduced the available slices for training the model to 1749 slices. This caused a reduction in the available data for training a deep learning model, which requires a considerable amount of training images to achieve good performance and prevent overfitting. Therefore, data augmentation was performed on the training data—using random (but realistic) modifications, such as image rotation and flipping, to broaden the diversity of the training set. This step increased the training data from 1749 to 5247 slices.

### Implementation of UNet with Various Selected Backbones

For the segmentation of the final pre-processed images, the use of UNet architecture was explored which consisted of a contracting path to capture context and a symmetric expanding way that enables precise localisation [17]. The convolutional neural network was implemented in Python using the Keras framework with TensorFlow as the backend [18]. For each developed model, extracted slices from the region of interest were used for the training and validation set, as described in the previous section.

The segmentation models for the left and right adrenal glands were developed using three backbones classification models as a feature extractor in addition to the default UNet architecture without any preference. The selected backbones are restnet34, vgg16, and inceptionv3. The models for the left and right adrenal glands were developed independently: a total of eight models. The implemented models have an encoder that serves to extract hierarchical, abstract features from the input image, preparing them for later stages of the network where they will be used for generating segmentation masks. The models’ decoder was responsible for upsampling and refining the feature maps from the encoder, combining them with skip connections to preserve spatial information, and ultimately producing a high-resolution segmentation mask that delineates objects or regions of interest in the input image. The combination of the encoder with the decoder, along with skip connections, enables the UNet to perform accurate pixel-wise segmentation tasks. To give the decoder component higher resolution features, the 2D UNet utilised skip connectors from layers of the same level. The networks were trained on mini-batches using the Adam optimiser, while rectified linear units (ReLU) and sigmoid were used as the activation functions. The training parameters were set to a batch size of 4, and the networks were trained for 100 epochs (as this was found to be the optimal value): early termination of training was not used, as overfitting was minimised by ensuring that the CT scans for all the slices in the training and validation set were mutually exclusive. The Jaccard index, also known as Intersection over Union, and the Jaccard similarity coefficient (originally coined coefficient de communauté by Paul Jaccard [19]) were used as the loss function.

Training, validation, and testing are carried out on a Windows 10 computer with an Intel Core i9 CPU and 128 GB of RAM. An NVIDIA GeForce RTX 3060 Ti with 8.0 GB of dedicated GPU memory and CUDA 9.0 was used for all experiments. Python 3.9 and anaconda3 were used for code implementation for all studies, including baseline techniques.

### Post-processing

The post-processing stage is a critical phase as it refines the segmentation results obtained from the developed model for the precise identification of the adrenal gland. This phase encompasses two pivotal stages, each designed to enhance the accuracy and coherence of the segmented regions. Firstly, TTA is implemented to augment the predictions made by the model, further enhancing its ability to capture intricate details and variations in the adrenal gland’s morphology. Subsequently, this stage involves a meticulous process of removing unconnected segmented gland regions, ensuring that the final output accurately delineates the boundaries of the adrenal gland.

### TTA

A data augmentation strategy was implemented to enhance performance and lower generalisation errors when training our neural network models for the segmentation of the adrenal glands. When making predictions using a fit model, the image data augmentation technique can also enable the model to generate predictions for numerous different versions of each image in the test dataset. TTA aims to make arbitrary changes to the test images. Averaging the predictions on the augmented images can improve the segmentation output. This step has been proven helpful in segmentation using deep learning [10, 20]. TTA was implemented using the following steps:

- Step 1: Predict the original test data.
- Step 2: On the original test data, reverse the order of the elements (left/right), and predict the output data.
- Step 3: On the original test data, reverse the elements' position (up/down), and predict the output data.
- Step 4a: On the original test data, reverse the elements' position (up/down).
- Step 4b: On the output of 4a, reverse the order of the elements (left/right) and predict the output data.
- Step 5: Find the average of all the predictions in steps 1 to 4.

### Removing Unconnected Segmented Gland

Peradventure there is missed segmentation of the adrenal gland outside the location of the supposed adrenal gland region; this step is created to remove such an error. The pixels of either right or left adrenal gland in a single CT scan will have a direct localised relationship with neighbouring pixels within that gland. Hence, any detected pixel outside this connection region can be considered an outlier and eliminated while producing the final segmentation. While there might not be a significant improvement in the segmented output or dice scores, this technique further reduces the false positives of the adrenal gland in pixelwise classification, a highly important measure when considering automated tumour segmentation.

### Evaluation Metrics

The proposed method and baseline approaches were evaluated using Dice similarity coefficient (DSC), precision, recall, and *F*1 score. The dice scores were calculated for each whole CT scan (per patient) rather than on individual slices, providing a comprehensive measure of DSC across the entire dataset, as conducted in Vinayahalingam et al. [21] and Xi et al. [22]. Additionally, precision, recall, and *F*1 score were calculated at the voxel level to provide a more detailed evaluation of segmentation performance across all the developed models.

### Experiments on Training and Testing on Separate Datasets

To evaluate the impact of dataset-specific characteristics on model performance, we conducted several experiments using the VGG16-based model. The results, presented in Table 1, highlight the performance differences when training on one dataset and testing on another, as well as when using a mixed dataset. When models were trained on one dataset and tested on a different dataset (Experiments 1 and 2), the dice scores were lower than when training and testing were performed on the same dataset (Experiments 5 and 6). This drop in performance suggests that differences in imaging protocols, scanner characteristics, and dataset distributions affect model generalisation across datasets. Experiments 3 and 4, where a combined dataset of AMOS and MICCAI was used for training, showed moderate improvements compared to cross-dataset testing but did not surpass the performance achieved when models were trained and tested on the same dataset. While training on the mixed dataset resulted in better generalisation, it did not yield the highest dice scores when evaluated on individual datasets. The best performance was observed when models were trained and tested on the same dataset (Experiments 5 and 6). This indicates that dataset-specific training allows the models to better learn the distributional properties of each dataset, leading to superior segmentation performance. Based on these findings, we adopted the strategy of training and testing models separately on each dataset for all model variants developed in this study. This approach ensures optimal performance while preserving the distinct characteristics of each dataset.

**Table 1:** Comparison of dice scores across different training and testing strategies

S/no	Experiments	Gland	Before	After
1	Trained model on AMOS dataset and tested on MICCAI	L.A.G	0.67	0.76
		R.A.G	0.65	0.74
2	Trained model on MICCAI dataset and tested on AMOS	L.A.G	0.61	0.70
		R.A.G	0.59	0.68
3	Trained model on a mixed dataset containing AMOS and MICCAI and tested separately on AMOS	L.A.G	0.80	0.89
		R.A.G	0.72	0.85
4	Trained model on a mixed dataset containing AMOS and MICCAI and tested separately on MICCAI	L.A.G	0.71	0.82
		R.A.G	0.69	0.77
5	Trained model on AMOS train data and tested on AMOS test data	L.A.G	**0.82**	**0.91**
		R.A.G	**0.75**	**0.90**
6	Trained model on MICCAI train data and tested on MICCAI test data	L.A.G	**0.78**	**0.90**
		R.A.G	**0.75**	**0.84**

## Results

In this section, the outcomes of the models’ segmentation are presented, emphasising the distinct models implemented for the right and left adrenal glands. This dual-model approach ensures precise delineation and comprehensive analysis of both anatomical structures in the context of test-time augmentation on CT images.

### Dice Scores for Right and Left Adrenal Glands

**Fig. 3:**
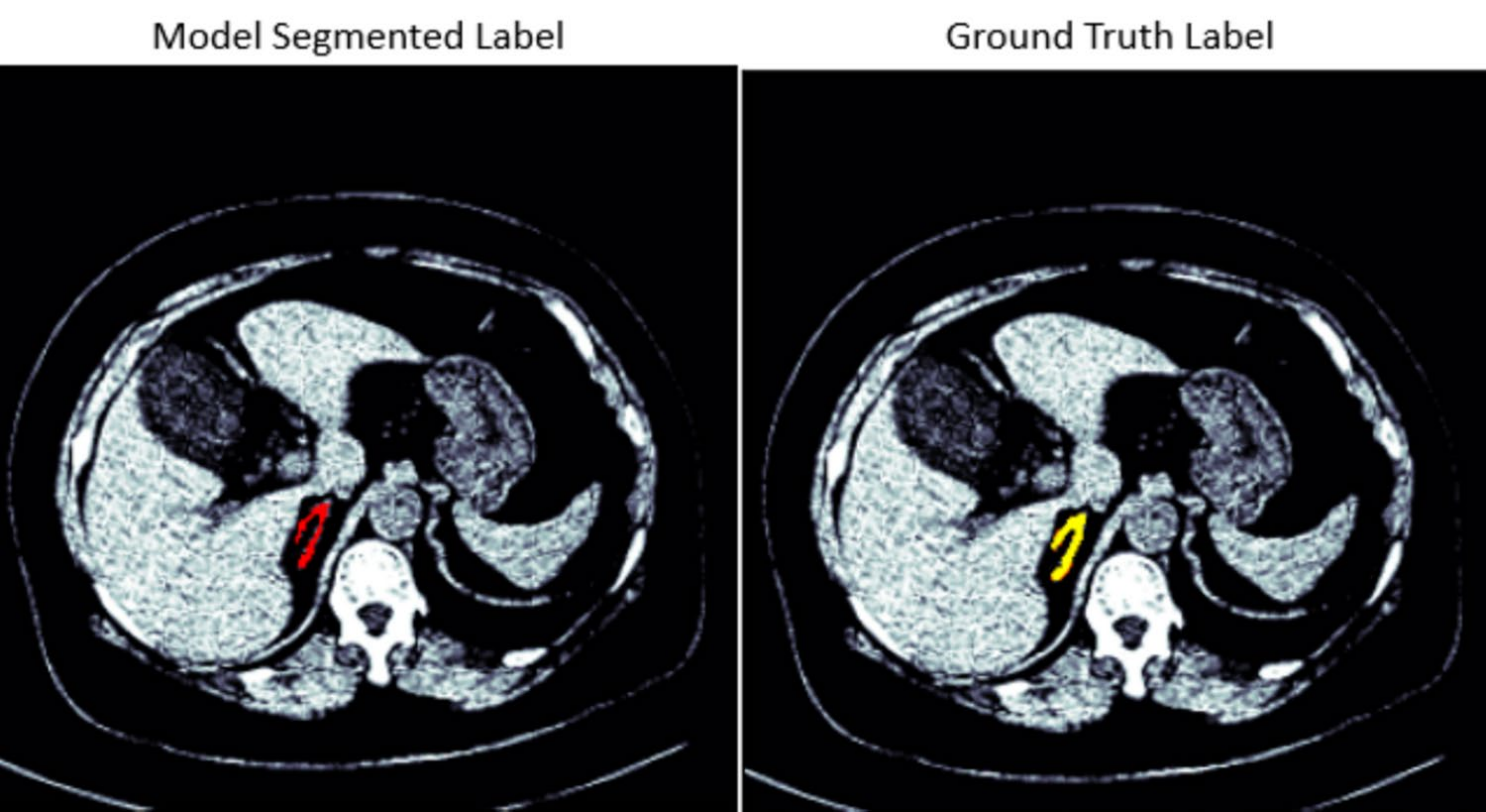
Segmentation output of a right adrenal gland from the AMOS dataset, showing the overlay of the mask on the CT scan. The mask predicted by the segmentation model is displayed in red, while the original ground truth label is displayed in yellow for comparison

**Fig. 4:**
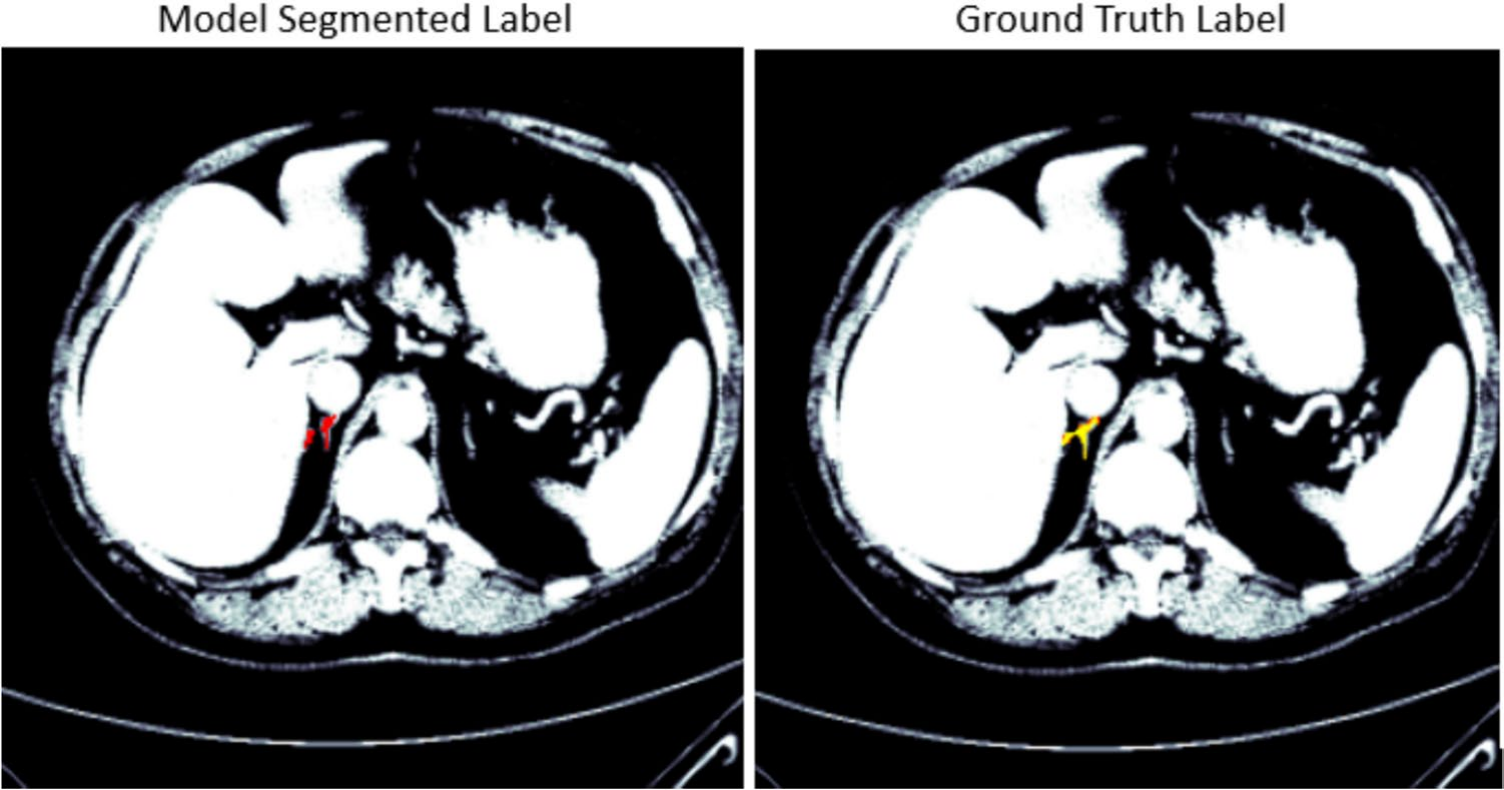
Segmentation output of a right adrenal gland from the MICCAI dataset, showing the overlay of the mask on the CT scan. The mask predicted by the segmentation model is displayed in red, while the original ground truth label is displayed in yellow for comparison

The UNet, restnet34, vgg16, inceptionv3, nnUNet, and TransUNet models were tested on 40 CT scans from the AMOS dataset that were separated prior to training the model. For the right adrenal gland, the 40 CT scans have 6481 slices in total where 453 slices contain the right adrenal gland. To show the effect of the post-processing techniques on all the developed models, the results were evaluated before the post-processing stage and after the post-processing, as shown in Tables 2 and 3. Before the post-processing was conducted, Table 2 shows the dice scores of 0.75, 0.79, 0.77, 0.80, 0.82, and 0.83 for VGG16, Resnet34, Inceptionv3, UNet, nnUNet, and TransUNet respectively. After the post-processing, the dice scores of 0.90, 0.88, 0.89, 0.90, 0.91, and 0.93 were recorded for VGG16, Resnet34, Inceptionv3, UNet, nnUNet, and TransUNet respectively. This respectively shows an increase of 20%, 11%, 16%, 13%, 11%, and 12%. The results from all the developed models using the MICCAI dataset showed a similar improvement with an increase of 12%, 15%, 15%, 10%, 14%, and 12% for VGG16, Resnet34, Inceptionv3, UNet, nnUNet, and TransUNet respectively. Figures 3 and 4 show the segmented model output of a slice and its ground truth label for a right adrenal gland from AMOS and MICCAI datasets respectively.

**Fig. 5:**
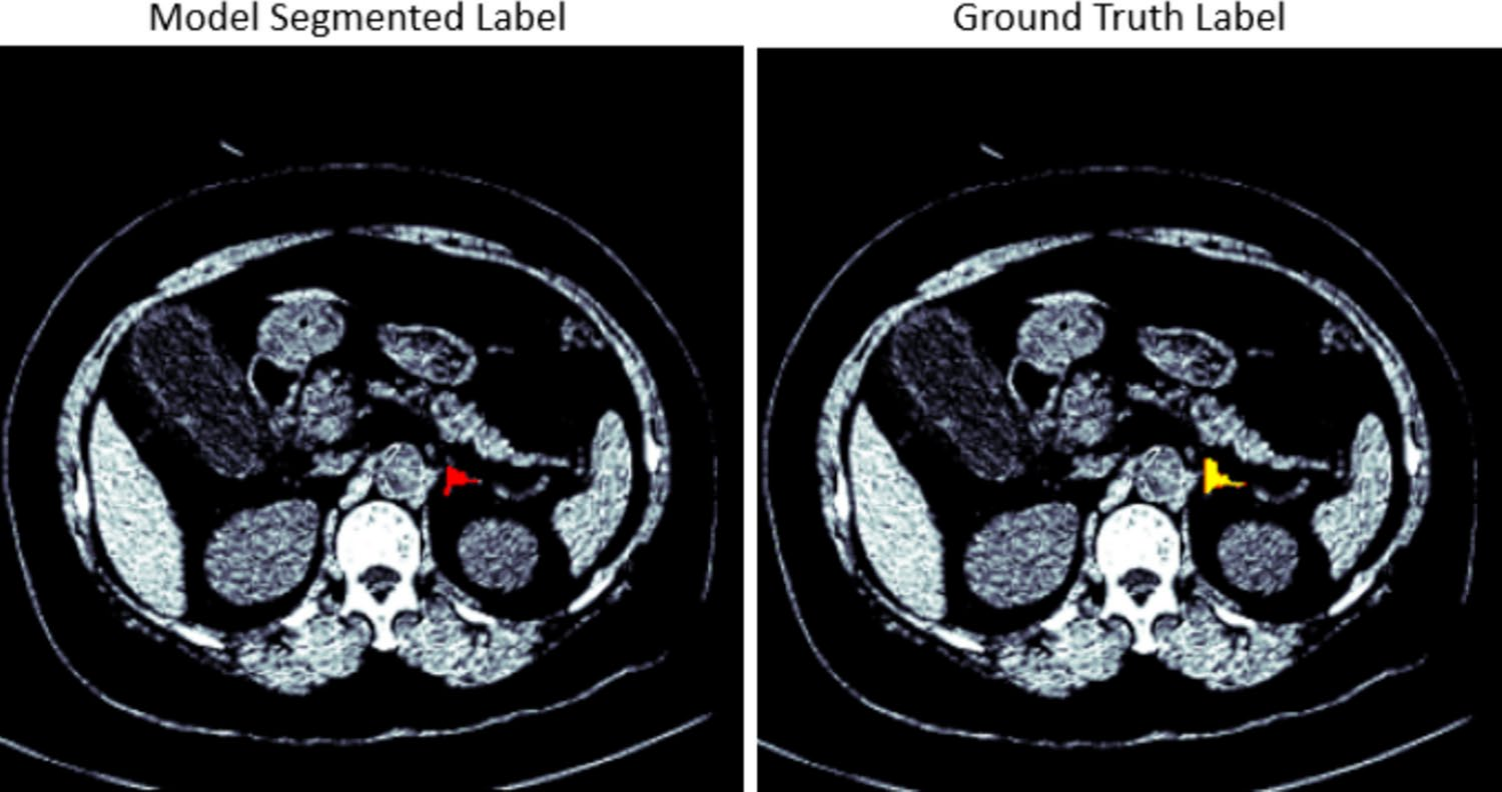
Segmentation output of a left adrenal gland from the AMOS dataset, showing the overlay of the mask on the CT scan. The mask predicted by the segmentation model is displayed in red, while the original ground truth label is displayed in yellow for comparison

**Fig. 6:**
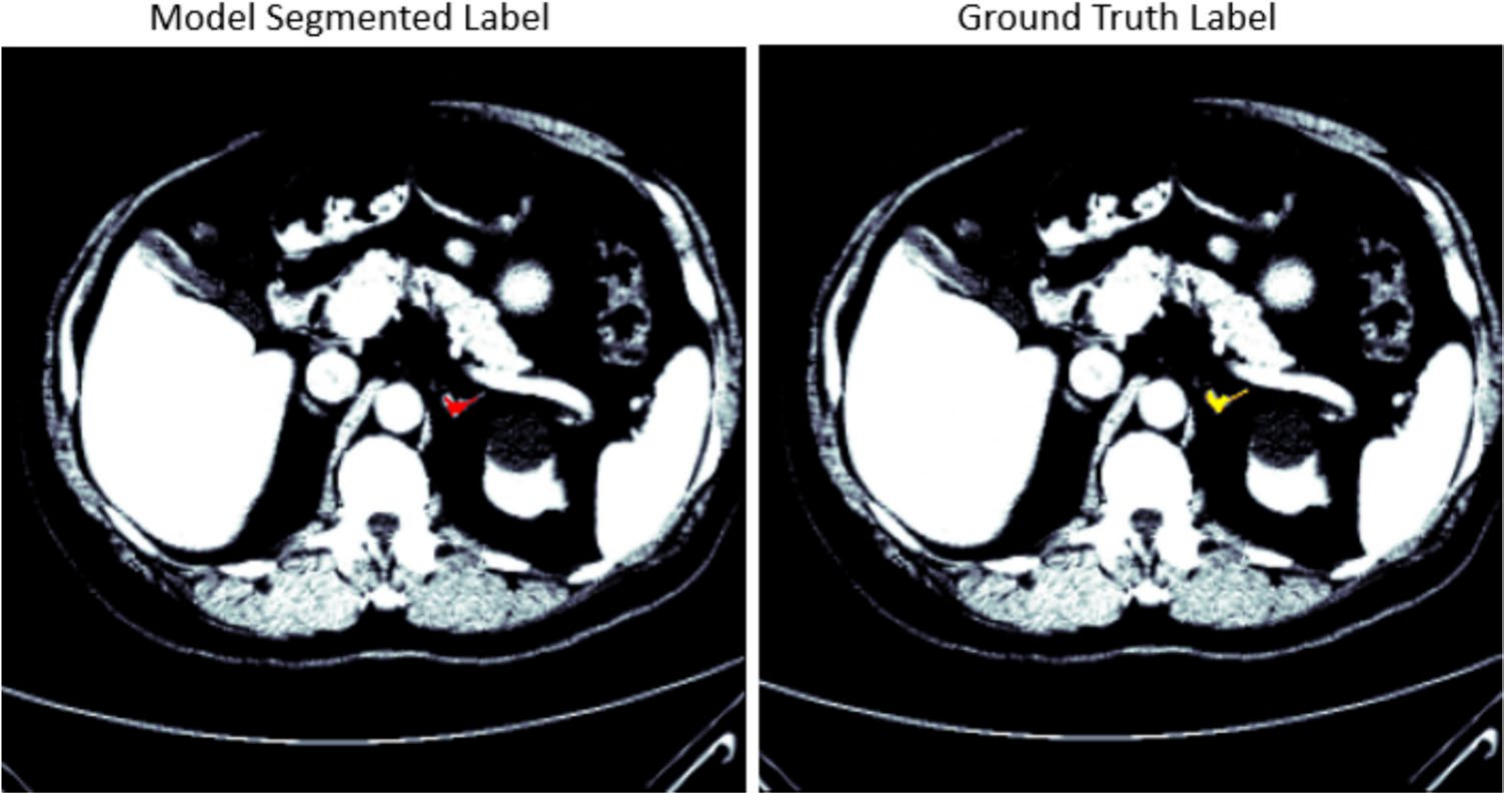
Segmentation output of a left adrenal gland from the MICCAI dataset, showing the overlay of the mask on the CT scan. The mask predicted by the segmentation model is displayed in red, while the original ground truth label is displayed in yellow for comparison

Of the 6481 slices for the 40 CT scans, there are 508 with the left adrenal gland and 5973 slices with no left adrenal gland. However, a similar effect of the post-processing technique was seen across all the developed models for the left adrenal gland. Before the post-processing, Table 2 shows the dice scores of 0.82, 0.76, 0.59, 0.72, 0.85, and 0.88 for VGG16, Resnet34, Inceptionv3, UNet, nnUNet, and TransUNet respectively. After the post-processing, as shown in Table 2, the dice scores of 0.91, 0.89, 0.85, 0.89, 0.92, and 0.92 for VGG16, Resnet34, Inceptionv3, UNet, nnUNet, and TransUNet respectively. This shows an increase of 11%, 17%, 48%, 19%, 8%, and 5% for VGG16, Resnet34, Inceptionv3, UNet, nnUNet, and TransUNet respectively. The results from all the developed models using the MICCAI dataset also showed a similar improvement with an increase of 15%, 47%, 11%, 12%, 13%, and 15% for VGG16, Resnet34, Inceptionv3, UNet, nnUNet, and TransUNet, respectively. Figures 5 and 6 show the segmented model output of a slice and its ground truth label for a left adrenal gland from AMOS and MICCAI datasets respectively.

**Table 2:** Dice score results before and after post-processing

Dataset	Gland	Model	Before	After	Actual diff	Increase rate (%)
AMOS	L.A.G	VGG16	0.82	0.91	0.09	**11**
		ResNet34	0.76	0.89	0.13	**17**
		InceptionV3	0.59	0.85	0.26	**44**
		UNet	0.72	0.89	0.17	**19**
		nnUNet	0.85	0.92	0.07	**8**
		TransUNet	0.88	0.92	0.04	**5**
	R.A.G	VGG16	0.75	0.90	0.15	**20**
		ResNet34	0.79	0.88	0.09	**11**
		InceptionV3	0.77	0.89	0.12	**16**
		UNet	0.80	0.90	0.10	**13**
		nnUNet	0.82	0.91	0.09	**11**
		TransUNet	0.83	0.93	0.10	**12**
MICCAI	L.A.G	VGG16	0.78	0.90	0.12	**15**
		ResNet34	0.53	0.78	0.25	**47**
		InceptionV3	0.74	0.82	0.08	**11**
		UNet	0.76	0.85	0.09	**12**
		nnUNet	0.80	0.90	0.1	**13**
		TransUNet	0.79	0.91	0.12	**15**
	R.A.G	VGG16	0.75	0.84	0.09	**12**
		ResNet34	0.75	0.86	0.11	**15**
		InceptionV3	0.78	0.90	0.12	**15**
		UNet	0.77	0.85	0.08	**10**
		nnUNet	0.81	0.92	0.11	**14**
		TransUNet	0.82	0.92	0.1	**12**

To validate the significance of the observed improvements in dice scores after post-processing, statistical analysis was conducted. Paired *t*-tests were performed to compare the dice scores before and after post-processing for each model. For the AMOS dataset, the left adrenal gland showed a statistically significant improvement with a *t*-statistic of − 3.89 and a *p*-value of 0.011, while the right adrenal gland demonstrated an even stronger improvement with a *t*-statistic of − 11.45 and a *p*-value of 0.000089. Similarly, for the MICCAI dataset, the left adrenal gland achieved a *t*-statistic of − 4.96 and a *p*-value of 0.004, and the right adrenal gland exhibited the most significant improvement with a *t*-statistic of − 16.92 and a *p*-value of 0.000013. These results confirm that the post-processing step significantly enhances segmentation performance across all models and datasets. Additionally, a one-way ANOVA was conducted for each gland type across the AMOS and MICCAI datasets to assess whether the improvements in dice scores varied significantly among the models. The analysis revealed no statistically significant differences in dice score improvements among the models for any gland type (*p* > 0.05). This indicates that the post-processing improvements were consistent across all models, demonstrating the robustness and generalisability of the proposed post-processing approach in enhancing segmentation performance.

### Precision for Right and Left Adrenal Glands

**Fig. 7:**
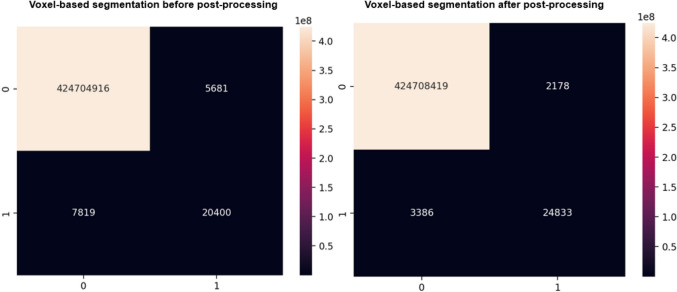
Confusion matrix for before and after post-processing techniques on the right adrenal gland using VGG16

**Fig. 8:**
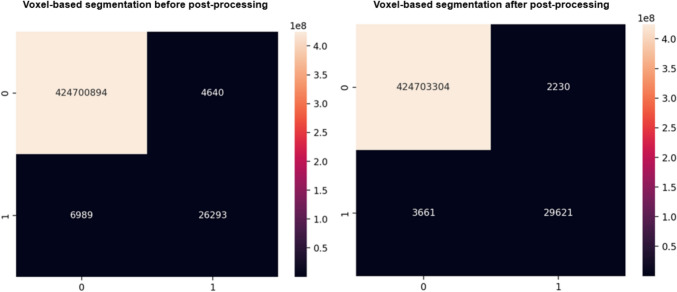
Confusion matrix for before and after post-processing techniques on the left adrenal gland using VGG16

The primary goal of this study is not to directly compare different models but to evaluate the effect of post-processing on the segmentation pipeline using selected architectures and backbones. While a significant increase in dice scores was observed across all models following post-processing, especially the TTA, a more detailed analysis was conducted to assess the impact at the voxel level, specifically on precision for the right and left adrenal glands. For the right adrenal gland, as shown in the confusion matrix in Fig. 7, post-processing drastically reduced the number of false positives from 5681 to 2178, representing a 62% reduction. While the number of false negatives also decreased significantly, from 7819 to 3386, the true positives increased from 20,400 to 24,833. This improvement elevated the model’s precision for the right adrenal gland from 78 to 92%, reflecting a 14% absolute increase. Similarly, for the left adrenal gland, as shown in the confusion matrix in Fig. 8, false positives decreased from 4640 to 2230 (a 52% reduction), while false negatives were reduced from 6989 to 3661. True positives correspondingly increased from 26,293 to 29,621. These changes led to an improvement in precision for the left adrenal gland from 85 to 93%, representing an absolute increase of 8%. As reflected in Table 3, similar improvements were observed across all models and datasets. For example, in nnUNet and TransUNet, precision consistently reached above 90% post-processing for both glands, demonstrating their robustness. Importantly, even in models with lower baseline performance, such as InceptionV3, the improvements were remarkable, with precision improving to clinically acceptable levels after post-processing.

**Table 3:** Voxel-based precision, recall, and *F*1 score results

			Precision		Recall		*F*1 score	
Dataset	Gland	Model	Before	After	Before	After	Before	After
AMOS	L.A.G	VGG16	0.85	0.93	0.79	0.89	0.82	0.91
		ResNet34	0.78	0.91	0.74	0.87	0.76	0.89
		InceptionV3	0.62	0.87	0.56	0.83	0.59	0.85
		UNet	0.75	0.91	0.69	0.87	0.72	0.89
		nnUNet	0.87	0.93	0.83	0.91	0.85	0.92
		TransUNet	0.90	0.93	0.86	0.91	0.88	0.92
	R.A.G	VGG16	0.78	0.92	0.72	0.88	0.75	0.90
		ResNet34	0.82	0.90	0.76	0.86	0.79	0.88
		InceptionV3	0.80	0.91	0.74	0.87	0.77	0.89
		UNet	0.82	0.92	0.78	0.88	0.80	0.90
		nnUNet	0.85	0.93	0.79	0.89	0.82	0.91
		TransUNet	0.86	0.94	0.80	0.92	0.83	0.93
MICCAI	L.A.G	VGG16	0.81	0.92	0.75	0.88	0.78	0.90
		ResNet34	0.60	0.81	0.47	0.75	0.53	0.78
		InceptionV3	0.77	0.85	0.71	0.80	0.74	0.82
		UNet	0.79	0.87	0.73	0.83	0.76	0.85
		nnUNet	0.84	0.91	0.76	0.89	0.80	0.90
		TransUNet	0.81	0.93	0.77	0.89	0.79	0.91
	R.A.G	VGG16	0.78	0.86	0.72	0.82	0.75	0.84
		ResNet34	0.77	0.88	0.73	0.84	0.75	0.86
		InceptionV3	0.80	0.92	0.76	0.88	0.78	0.90
		UNet	0.79	0.87	0.75	0.83	0.77	0.85
		nnUNet	0.83	0.93	0.79	0.91	0.81	0.92
		TransUNet	0.83	0.95	0.81	0.89	0.82	0.92

**Fig. 9:**
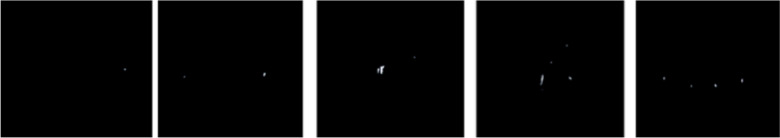
Examples of false positive slices

The remaining false positive segmented glands were found to be in the region of the adrenal gland; an example of one of the false positive segmented glands is shown in Fig. 9. This further highlights the robustness and effectiveness of the segmentation model. It indicates that while some false positives were present, they were not entirely erroneous. Instead, they were located in close proximity to the actual adrenal gland, suggesting that the model demonstrated a high degree of accuracy in identifying relevant anatomical structures. This information is crucial in a medical context, as it implies that even the misidentified regions were biologically relevant, minimising the risk of potentially overlooking clinically significant features. This can greatly enhance the overall utility and reliability of the segmentation results for medical practitioners and researchers.

### Comparison of Dice Scores Across Models and Related Works

The bilateral average dice scores for both the right and left adrenal glands achieved by the proposed models were 89% for VGG16, 85% for ResNet34, 87% for InceptionV3, 87% for UNet, 91% for nnUNet, and 92% for TransUNet. When compared to related works on adrenal gland segmentation, all the developed models outperformed the approaches reported by Zhou et al. [5] and Tang et al. [4], which achieved dice scores of 35.48% and 73.48%, respectively. Additionally, the VGG16, nnUNet, and TransUNet models surpassed the performance of Luo et al. [3], which reported a dice score of 87.42%. These findings underscore the effectiveness and superiority of the proposed segmentation pipeline in improving adrenal gland segmentation accuracy.

## Discussion

**Fig. 10:**
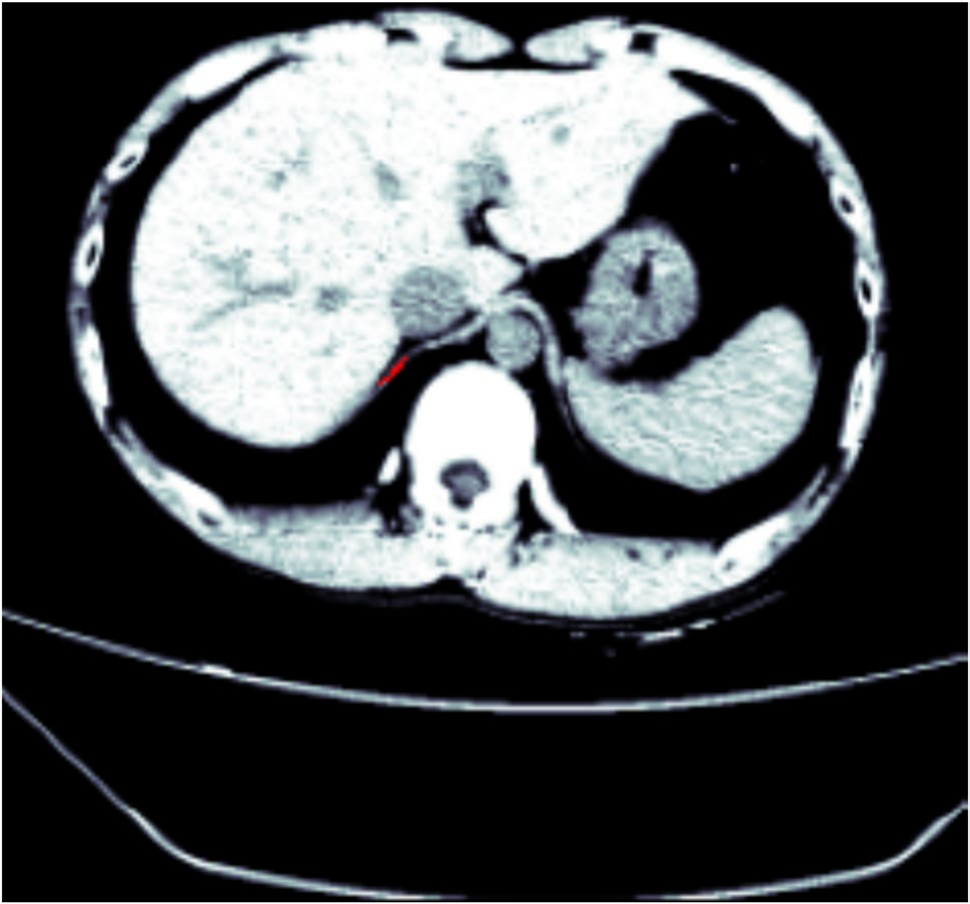
Example of a false positive of the right adrenal gland, located in close proximity to the actual location of the adrenal gland

While some literature focuses on new architectures, algorithms, and models, the focus of this work is on principles, guidelines, approaches, and steps in improving segmentation results using existing architectures. The efficiency and effectiveness of our proposed pipeline are evidenced in our presented results. Hence, it is necessary to state that this paper aims to improve the output of any given segmentation model using our proposed pipeline. In the results, TTA reduces the false positive and increases the precisions of all the developed models. In addition, the elimination of all isolated adrenal glands from each CT scan ensures that the segmented adrenal glands are in the exact location. For further clarification, the missed segmented adrenal glands were visualised and seen to be around the actual region of the adrenal glands. An example of a missed segmented right adrenal gland is shown in Fig. 10. Before the post-processing stage, the results showed that some adrenal glands were detected outside the left and right adrenal glands region and this affected the generalisation results. One ideal approach to ensuring model generalisation is to increase the number of images in the training set. However, this necessitates more labour-intensively segmented CT scans, which are not always readily available. Post-processing shows that using a TTA technique and removing all unconnected adrenal glands can considerably improve the generalisation results.

To further verify the importance of the pre-processing technique, especially the use of only slices that contain the adrenal gland for training purposes only, a model was trained using all the slices in the CT scans, and the model was unable to learn the features of the adrenal gland. This proved the importance of removing slices with no adrenal gland for the training in the pipeline.

A limitation of this study is the reliance on a 2D segmentation approach, which, while effective with the implemented preprocessing and post-processing techniques, may not fully capture the spatial context available in 3D CT images. Future work should explore 3D segmentation models to leverage volumetric information and evaluate their performance in comparison to the current pipeline.

## Conclusions

This study presents a novel and highly effective pipeline for adrenal gland segmentation from CT images, addressing key challenges associated with their small size, variable shape, and proximity to surrounding tissues. By combining pre-processing techniques with a sophisticated post-processing strategy, including test-time augmentation and removing unconnected segments, our approach demonstrates significant improvements in segmentation accuracy across multiple models. The proposed pipeline is adaptable to various UNet backbones. It outperforms existing state-of-the-art methods, achieving up to a 44% improvement in dice scores and a reduction of false positives by 40%. This work marks an important step forward in the field, offering a robust solution that can be readily implemented in clinical settings, thereby advancing the accuracy and reliability of adrenal gland segmentation in medical imaging.

## Data Availability

The data are available at 10.5281/zenodo.7155725 and Multi-Atlas Labeling Beyond the Cranial Vault—Workshop and Challenge—syn3193805—Wiki (synapse.org).
